# Structural color-based microneedle patch with NIR-triggered on-demand drug release for intelligent treatment of infected wounds

**DOI:** 10.1186/s12951-026-04730-6

**Published:** 2026-06-27

**Authors:** Sijia Huang, Menglan Yang, Chen Ding, Wenfang Du, Shengyuan Yang, Qiang Xi, Fubing Xiao

**Affiliations:** 1 Hunan Prevention and Treatment Institute for Occupational Diseases, Affiliated Prevention and Treatment Institute for Occupational Diseases, School of Public Health, Hengyang Medical School, University of South China, Changsha, 410007 China; 2 School of Chemical and Environmental Engineering, Hunan Institute of Technology, Hengyang, 421002 China; 3 Laboratory for the Development and Application of New Technologies in the Diagnosis and Treatment of Chemical Poisoning, Clinical Medical Research Center for Occupational Diseases, Changsha, 410007 Hunan Provincial China

**Keywords:** Hydrogel microneedles, Antibacterial/antioxidant properties, Photothermal therapy, Visual feedback, Infected wound healing

## Abstract

Bacterial-infected wounds present a critical clinical challenge due to persistent inflammation, impaired angiogenesis, and the lack of real-time treatment monitoring. To address these issues, we developed an innovative colloidal crystal hydrogel microneedle (CT MNs) patch based on CuS nanoparticles (CuS NPs) and tannic acid-berberine nanoparticles (TB NPs). This system integrates synergistic antibacterial/antioxidant therapy with structural color-based drug release self-reporting capability. Specifically, the CT MNs combine photothermal CuS NPs for near‑infrared (NIR)-triggered hyperthermia and temperature‑controlled drug release, the release of TB NPs for potent antibacterial/antioxidant activities, and the color changes of the inverse opal colloidal crystal for real‑time monitoring of drug release. In vitro assays demonstrated strong antibacterial effects (95.65% and 95.92% against *E. coli* and *S. aureus*, respectively) and outstanding ROS‑scavenging capacity (92.93% for DPPH and 97.36% for ABTS⁺). In infected rat models, the CT MNs + NIR group achieved rapid wound closure (94.41% by day 10), significantly outperforming the controls. This accelerated healing was attributed to the synergistic effects of photothermal ablation, TB NPs-mediated bacterial clearance, and ROS-scavenging-driven inflammation alleviation. Therefore, this work presents a theranostic dressing that merges targeted combination therapy with non‑invasive optical monitoring of drug release, offering a promising strategy for intelligent wound management.

**Graphical Abstract:**

**Figure:**
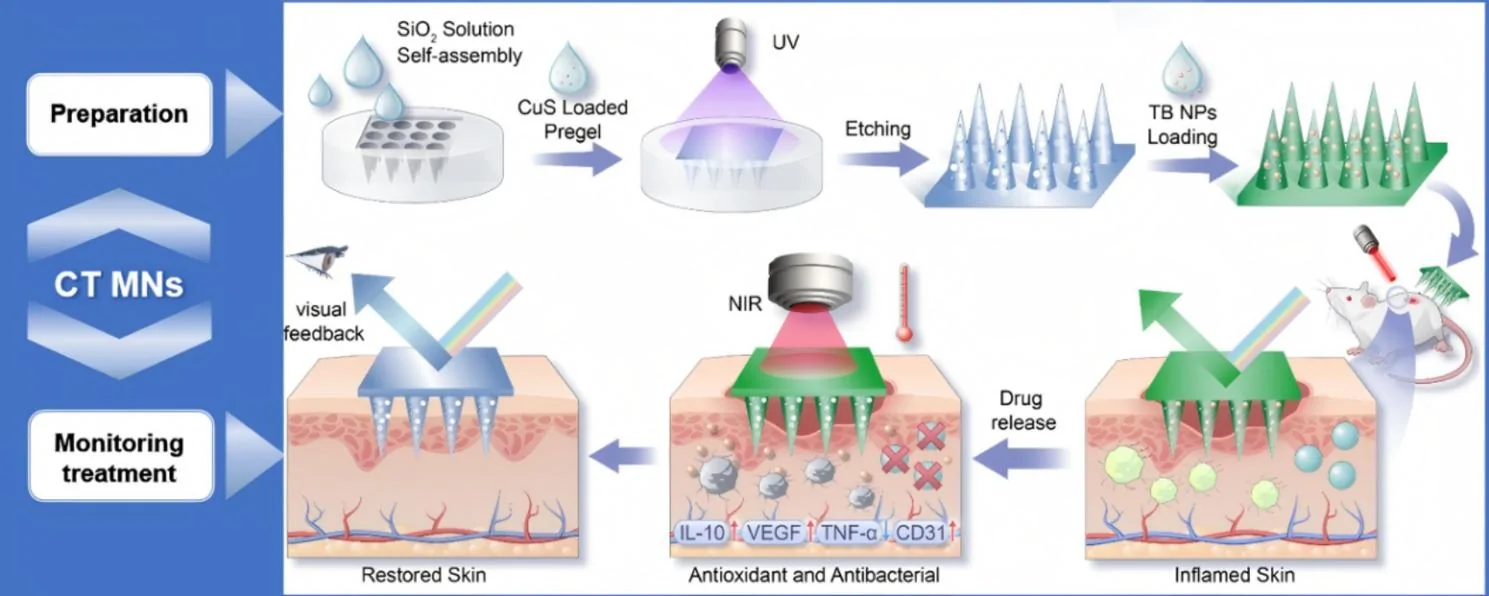

**Supplementary Information:**

The online version contains supplementary material available at https://doi.org/10.1186/s12951-026-04730-6.

## Introduction

Skin injuries, resulting from external trauma or underlying diseases, are highly susceptible to bacterial infection, leading to severe health issues and substantial socioeconomic burdens. Wound healing comprises a complex, multi-stage physiological process that includes inflammation, proliferation, and tissue remodeling [1]. Disruption of any stage can delay recovery. Bacterial infection represents a primary impediment to healing, not only by provoking localized inflammation but also by potentially progressing to systemic infections that threaten patient survival [2, 3]. Moreover, the treatment of infected wounds faces challenges such as antibiotic resistance and inefficient drug delivery efficiency, imposing significant strain on public health systems [4]. Conventional treatments, including injections, oral administration, bandages, and gauze, exhibit limitations in meeting the multifaceted demands of infected wound management, such as concurrent antibacterial action, inflammation suppression, and immunomodulation. They are also hindered by suboptimal drug delivery efficiency, poor control over local drug concentrations, and a tendency to induce drug resistance [5]. Therefore, developing efficient, safe therapeutic materials and novel strategies for treating bacterial-infected wounds is of great practical significance for public health.

To address the complex stress response induced by bacterial infections, the biomedical field has innovatively developed multifunctional dressings materials such as porous foams [6], membranes [7], and hydrogels [8–10]. Among these, hydrogels are widely regarded as superior wound dressings due to their excellent biocompatibility and functional versatility. Hydrogel microneedles (MNs), as an emerging transdermal drug delivery system, have shown great potential in wound treatment [11, 12]. Compared to traditional methods, MNs offer advantages including minimal invasiveness, ease of application, and capacity for localized, controlled drug release, making them an ideal carrier for transdermal delivery. Unlike conventional hydrogels blocked by necrotic tissue and biofilms, MN tips penetrate the barrier and deliver drugs directly into the dermis [13–17]. However, most existing hydrogel patches lack the ability to continuously and effectively monitor wound status, making it difficult to achieve on-demand regulation of wound inflammation and real‑time indication of drug release. Thus, the development of an intelligent hydrogel‑based sensing patch capable of monitoring drug release in real time to assess healing progress is urgently needed.

Incorporating stimulus‑responsive elements into MNs allows for controlled drug release triggered by external triggers including light [18], magnetic fields [19], and temperature [20–22]. Among these, 808 nm near-infrared (NIR) light has been widely utilized in photothermal antibacterial therapy due to its advantages of deep tissue penetration and minimal damage to normal tissues [23–25]. Current sensing approaches for MNs mainly rely on electrochemical [26–28] or fluorescent techniques [29–32]. Although significant progress has been made, these sensing methods often involve complex fabrication and depend on bulky instrumentation. In contrast, structural color, generated through light interference within periodic ordered structures [33, 34], has shown great potential for biomedical sensing and display. The inverse opal colloidal crystal architecture, an ordered interconnected porous framework, offers a high specific surface area and tunable structural color [35–40]. This distinctive structure enables it to function both as an intelligent self-reporting platform and a delivery carrier for various active components. Therefore, the integration of an inverse opal colloidal crystal structure into MNs is expected to open new avenues for multi-stage wound healing with real-time feedback.

**Fig. 1:**
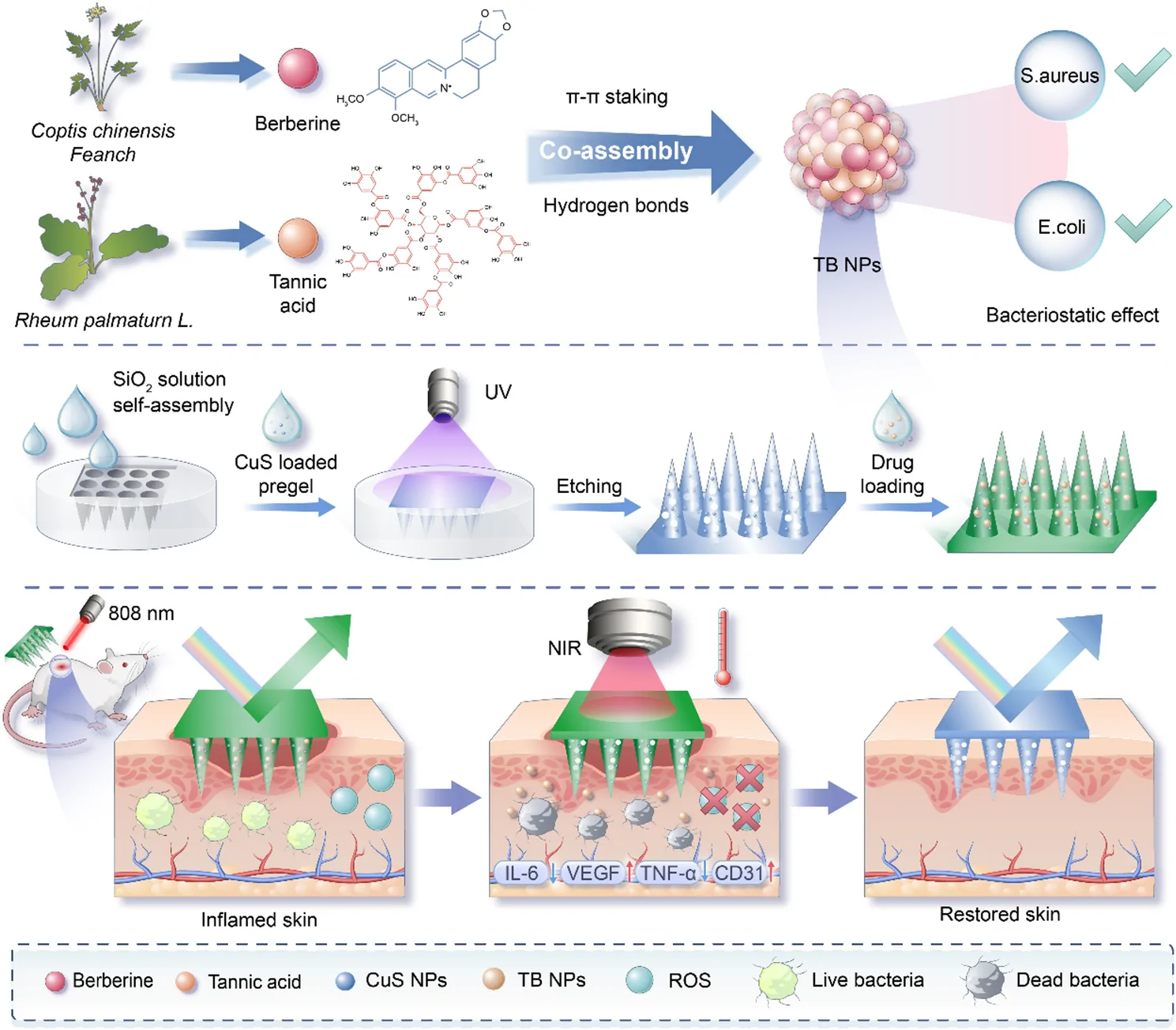
Schematic CT MNs designed for infected wound therapy

This study presents structural color-based microneedles (CT MNs) for intelligent management of infected wounds by integrating photo‑responsive copper sulfide nanoparticles (CuS NPs) and tannic acid‑berberine nanoparticles (TB NPs) into an inverse opal colloidal crystal hydrogel (Fig. 1). The CT MNs are fabricated through a three‑step process: (1) polymerization of a CuS NP‑PEGDA mixture in a colloidal crystal template MN mold to form colloidal crystal MNs, (2) etching to obtain an inverse opal MNs (IOMNs), and (3) loading the pores with agarose‑encapsulated TB NPs. Upon application, CuS NPs generate localized heat under 808 nm NIR irradiation, which accelerates the release of TB NPs to enable synergistic antibacterial action via photothermal ablation and direct nanoparticle activity. The released TB NPs further mitigate oxidative stress by eliminating reactive oxygen species (ROS), thereby improving the wound microenvironment for tissue regeneration. As TB NPs are released, hydrogel contraction alters the effective refractive index of the colloidal crystal lattice, producing a measurable blue shift in structural color that serves as a built‑in optical self‑reporting mechanism for real‑time, non‑invasive drug‑release monitoring. Thus, this work develops a multifunctional theranostic platform that integrates targeted antibacterial/antioxidant therapy, enhanced wound regeneration, and a promising optical self‑reporting capability into a precise strategy for intelligent wound management.

## Materials and methods

### Materials

Polyvinylpyrrolidone (PVP), tetraethyl orthosilicate (TEOS), sodium sulfide nonahydrate (Na_2_S·9H_2_O), tannic acid (TA), 2-hydroxy-2-methylpropiophenone (HMPP), and poly(ethylene glycol) diacrylate (PEGDA, Mn ≈ 700) were obtained from Shanghai Aladdin Biochemical Technology Co., Ltd. (Shanghai, China). Berberine was supplied by Shanghai Macklin Biochemical Technology Co., Ltd. (Shanghai, China). DMEM medium, fetal bovine serum (FBS), and phosphate buffered saline (PBS, pH 7.4) were supplied by Thermo Fisher Scientific (MA, USA). Live/dead cell staining kit and Cell Counting Kit-8 (CCK-8) were acquired from Beyotime Biotechnology (Shanghai, China). NIH 3T3 fibroblast cells were supplied by Cell Bank of the Chinese Academy of Sciences (China). *Escherichia coli* (*E. coli*, ATCC 25922) and *Staphylococcus aureus* (*S. aureus*, ATCC 29213) were procured from the China General Microbiological Culture Collection Center (Beijing, China). Adult male Sprague Dawley (SD) rats (210–250 g) were obtained from Hunan Slake Jinda Laboratory Animal Co., Ltd. (Changsha, China). All animal procedures adhered to institutional guidelines and were approved by the Laboratory Animal Ethics Committee of University of South China.

### Preparation of CT MNs

A colloidal crystal template was first prepared by injecting a 5% (w/v) SiO_2_ ethanol solution (sonicated for 5 min) into hydrophilically treated polydimethylsiloxane (PDMS) MN molds. After vacuum degassing and drying, the template was infiltrated with a precursor gel containing PEGDA (40%, v/v), CuS NPs (5 mg/mL), and photo-initiator HMPP (1%, v/v). The gel was then photopolymerized under UV light (365 nm, 10 mW/cm^2^) for 2 min to form colloidal crystal MNs. Subsequently, the solidified MNs were peeled off and etched overnight in 4% HF to remove the SiO_2_ template, yielding IOMNs, followed by thorough rinsing with deionized water. For drug loading, 2 mg/mL TB NPs were first homogeneously dispersed in an agarose solution (2%, w/v) by heating to 80 °C with stirring. After cooling the solution to 60 °C, the MN patch was immersed and subjected to vacuum degassing to ensure thorough infiltration of the colloidal crystal pores. Finally, the patch was removed and allowed to gel at room temperature. After cooling and gelation, the drug-loaded CT MNs were obtained. The resulting MN tips measured approximately 830 μm in height and 360 μm in base diameter. Optical microscopy images were captured using a CCD-equipped microscope, and diffraction spectra were acquired with a fiber-optic spectrometer (NOVA, Ideaoptics).

### NIR photothermal performance characterization

The photothermal response of MNs was assessed by irradiating samples containing different CuS NP concentrations (0–10 mg/mL) with an 808 nm NIR laser (2.0 W/cm^2^) for 5 min, while temperature changes were monitored in real time using an infrared thermal camera (UTi206B, UNI-T, China). Photothermal stability was evaluated over five repeated ON/OFF cycles (5 min irradiation followed by 5 min cooling) for MNs containing 5 mg/mL CuS NPs. Additionally, the power‑dependence of the photothermal effect was examined by irradiating MNs at varying laser power densities and recording the corresponding temperature profiles.

### Drug release behavior

The TB NPs loading amount was determined by the gravimetric method. Briefly, blank IOMNs were weighed (*W₀*), infiltrated with 2 mg/mL TB NPs/agarose solution, and re-weighed after gelation (*W₁*). The loading amount was calculated as (*W₁* – *W₀*) × C_TB_ / ρ_solution_ × 1000, where C_TB_ = 2 mg/mL and ρ_solution_ ≈ 1 g/mL. After calculation, the TB NPs loading amount of CT MNs was about 0.1 mg.

Drug release kinetics were evaluated by immersing the CT MNs in PBS (10 mL, pH 7.4) at 37 ℃ under two conditions, namely with and without NIR irradiation (808 nm, 2 W/cm^2^). At different time points, 200 µL aliquots of the release medium were sampled and replenished with an equal volume of fresh PBS to preserve sink conditions. The release of TB NPs from the CT MNs was monitored at 273 nm by UV-Vis spectrophotometry and quantified using a standard calibration curve (Fig. S1). Diffraction spectra of the MNs were recorded before and after the release period using a fiber-optic spectrometer (*n* = 3, biological replicates).

**Fig. 2:**
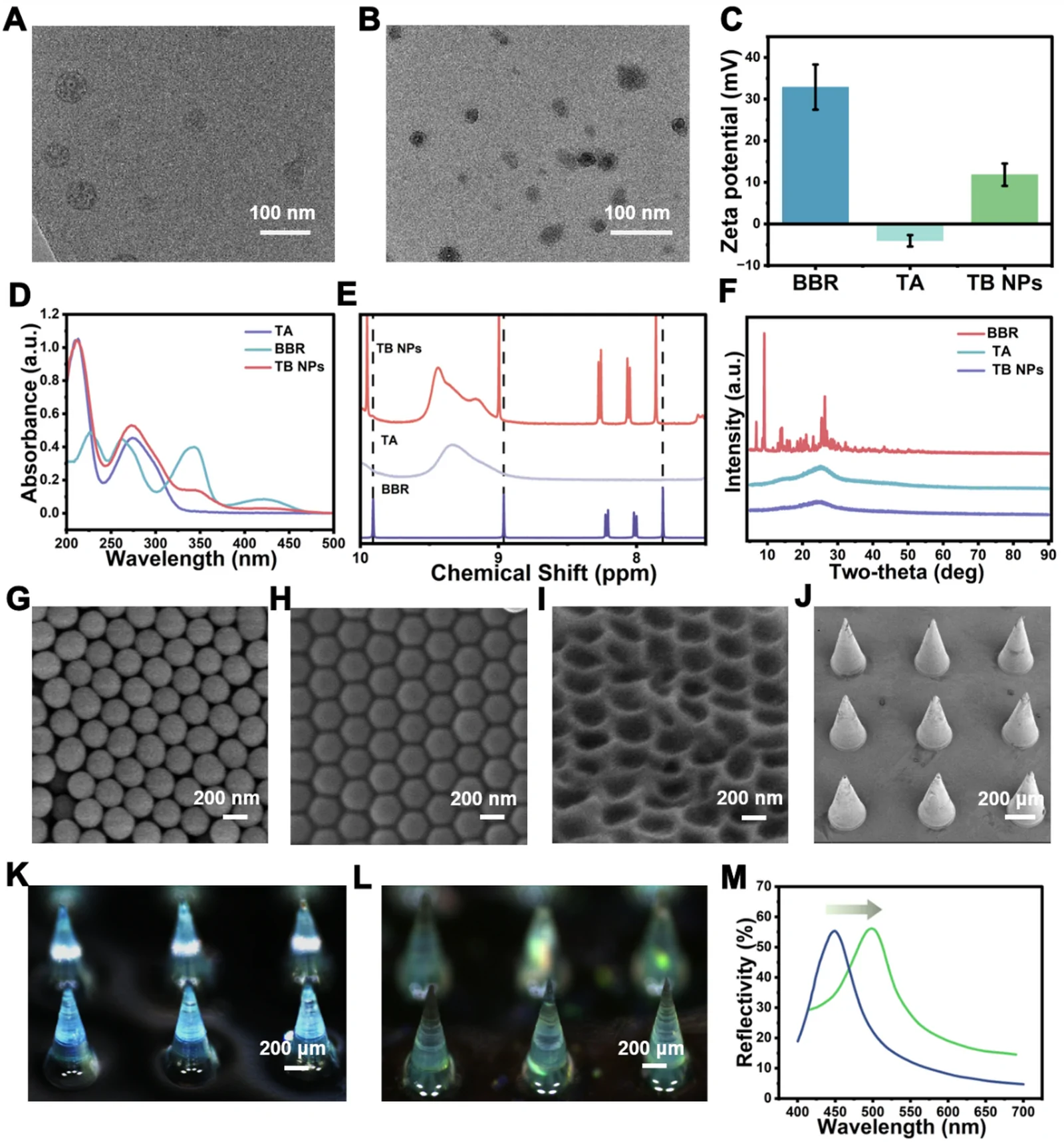
Characterization of MNs. TEM images of (**A**) CuS NPs and (**B**) TB NPs; (**C**) Zeta potential, (**D**) UV-Vis absorption spectra, (**E**) ^1^H NMR spectra, and (**F**) XRD pattern of TA, BBR, and TB NPs; SEM images of (**G**) colloidal crystal template, (**H**) colloidal crystal MNs, (**I**) IOMNs, and (**J**) IOMN tips; Optical images of (**K**) colloidal crystal MN tips and (**L**) IOMN tips; (**M**) Diffraction spectra of MNs before and after etching

The UV-Vis detection method for TB NPs was validated. As shown in Fig. 2D, the UV-Vis spectrum of intact TB NPs exhibited a characteristic peak at 273 nm, which is distinct from those of free berberine (BBR, 261 nm) and free tannic acid (TA, 275 nm). Release samples showed an identical peak at 273 nm without detectable free BBR or TA peaks, while blank hydrogel and agarose extracts exhibited negligible absorbance at 273 nm, confirming the specificity of this detection method (Fig. S1B).

### In vitro antibacterial experiments

The antibacterial efficacy was investigated against *E. coli* and *S. aureus* using a suspension co-culture method. Four experimental groups were established: a PBS-treated negative control, PEGDA MNs (without active components), CuS MNs (CuS NPs-loaded MNs), and CT MNs (MNs co‑loaded with CuS NPs and TB NPs). Each group was further divided into two subgroups: with or without 808 nm NIR light exposure. Each type of MNs was co-cultured with 2 mL of bacterial suspension (~ 10^5^ CFU/mL) in LB broth at 37 °C with shaking for 18–24 h. For the optical density measurement, the optical density at 600 nm (OD_600_) of the culture was measured spectrophotometrically. The antibacterial rate (%) was calculated as (*OD*_*control*_ − *OD*_*test*_) / *OD*_*control*_ × 100%, where *OD*_*control*_ and *OD*_*test*_ are the OD_600_ values of the PBS control group and the test group, respectively (*n* = 3, biological replicates). For the colony-forming unit (CFU) counting assay, the bacterial suspension was serially diluted to an appropriate concentration range and plated on LB agar plates. Following incubation at 37 °C for 24 h, colonies were counted. The antibacterial rate (%) was determined as (*C*_*0*_ − *C*_*s*_) /*C*_*0*_ × 100%, where *C*_*0*_ and *C*_*s*_ represent the viable bacterial concentrations (CFU/mL) of the PBS control group and the experimental group, respectively (*n* = 3, biological replicates). In addition, bacterial suspensions (20 µL each) were spread onto LB agar plates, followed by incubation at 37 °C for 24 h. The resulting colonies were then photographed for subsequent assessment of antibacterial activity.

### Cytotoxicity evaluation

Cytotoxicity was assessed using a CCK‑8 assay with NIH/3T3 fibroblasts. Cells were treated with extract solutions (1 mg/mL) from PEGDA MNs, CuS MNs, or CT MNs, while pure culture medium served as the control. After incubating the cells for 12, 24, and 48 h, cell viability was assessed via CCK‑8 assay. At each time point, a mixture of 10 µL CCK‑8 and 90 µL culture medium was added to each well. After 30 min incubation, the absorbance at 450 nm (OD_450_) was measured using a microplate reader (VersaMax, VWR International, USA). Cell viability was calculated as (*OD*_*test*_ – *OD*_*blank*_) / (*OD*_*control*_ − *OD*_*blank*_) × 100%, where *OD*_*tes*t_, *OD*_*blank*_, and *OD*_*control*_ represent the OD_450_ values of test group, the blank (no cells), and control group, respectively (*n* = 3, biological replicates). In parallel, live/dead staining was performed using Calcein‑AM (green, live cells) and propidium iodide (red, dead cells) after the same time points, with cell status visualized by fluorescence microscopy (Nikon DS-Ri2, Japan).

### Wound healing evaluation

A full‑thickness skin infection model was established in SD rats using a 10 mm circular punch biopsy tool, followed by inoculation with *S. aureus* (1 × 10^8^ CFU/mL, 30 µL). Infected animals were randomly allocated into five groups by random number table, with 4 SD rats in each group (*n* = 4): PBS control, PEGDA MNs, CuS MNs, CT MNs, and CT MNs + NIR. Dressings were changed daily during the two‑day treatment period. For the CT MNs + NIR group, a 5 min NIR irradiation was applied following the attachment of the MNs. On day 3, wound exudate was collected and cultured on agar plates to assess bacterial presence. Wounds were photographed on days 0, 1, 3, 5, 7, and 10, and wound area was quantified using ImageJ software. The wound closure rate was calculated as the equation: Wound closure rate (%) = (*S*_*0*_ − *S*_*n*_) / *S*_*0*_ × 100%, where *S*_*0*_ and *S*_*n*_ are defined as the initial wound area and the area on day *n*.

## Results and discussion

### Preparation and characterization of MNs

This study developed a multifunctional photothermal MN system by integrating CuS NPs and self-assembled TB NPs into an inverse opal hydrogel structure. The CuS NPs, synthesized with polyvinylpyrrolidone as a stabilizer, exhibited a spherical morphology with a hydrodynamic diameter of 52.48 nm (Fig. 2A, S2A). The TB NPs were formed through a carrier‑free co‑assembly of berberine (BBR) and tannic acid (TA), showing a diameter of 53.41 nm, a polydispersity index of 0.0969, and a zeta potential of + 11.83 mV (Fig. 2B, C, S2B), which indicates favorable colloidal stability. The co‑assembly of TB NPs was characterized by UV‑Vis spectroscopy. As shown in Fig. 2D, characteristic absorption peaks of BBR and TA were observed at 226/261/343 nm and 213/275 nm, respectively. The UV‑Vis spectra of TB NPs exhibited absorption features of both BBR and TA, indicating the successful incorporation of the two components. Notably, an absorption peak of 273 nm was evident in TB NPs compared with free BBR (261 nm) and TA (275 nm), suggesting the formation of non‑covalent interactions within the assembled system. Further structural analysis using ¹H NMR revealed significant chemical shifts in the isoquinoline ring protons of BBR (from 9.907, 8.961, and 7.808 ppm to 9.951, 8.996, and 7.860 ppm, respectively), supporting the formation of π–π stacking between BBR and TA (Fig. 2E, S2C). The XRD patterns of the nanoparticles showed no sharp diffraction peaks (Fig. 2F), confirming their amorphous nature—a structural feature that is advantageous for facilitating drug release.

IOMNs were fabricated by templating against a vertically deposited SiO_2_ colloidal crystal, followed by infiltration with a PEGDA‑based pre‑gel containing CuS NPs, photopolymerization, and etching with HF. The resulting porous scaffold was subsequently loaded with TB NPs via an agarose solution. SEM imaging demonstrated a highly ordered colloidal crystal architecture in both the colloidal crystal template and the final hydrogel network, along with well‑defined and mechanically intact MN tips (Fig. 2G‑J, S3). EDS elemental mapping confirmed that the CuS NPs remained uniformly distributed within the matrix after the etching process (Fig. S4).

The mechanical properties of the MNs were characterized to ensure their functionality for skin penetration. According to the stress-displacement curve, a single MN needle was capable of withstanding over 0.14 N (Fig. S5A), indicating sufficient strength for skin penetration. This was further validated in vivo by inserting the MNs into SD rat skin under appropriate force. Following insertion, clear micropores were observed on the skin surface (Fig. S5B, C), and histological sections verified epidermal penetration (Fig. S5D, E). These results verify that the MNs possess the necessary mechanical robustness for effective transdermal delivery in wound healing experiments.

Optically, the colloidal crystal MNs and IOMNs exhibited vivid structural colors (Fig. 2K, L). A distinct color shift was observed after removing the template, accompanied by a corresponding blue shift in the diffraction wavelength (Fig. 2M). This confirms that the ordered architecture of the inverse opal colloidal crystal was successfully fabricated and well preserved. According to Bragg’s law, the wavelength (λ) of the main reflection peak under normal incident light can be described by the relationship *λ* = 1.633·*d*·*n*_average_, where *d* is the center-to-center distance between adjacent nanoparticles or nanopores, and *n*_average_ is the average refractive index of the colloidal crystal structure [35]. Consequently, the diffraction wavelength is determined by refractive index. This measurable optical response provides the foundation for real‑time monitoring, as the release of TB NPs from the pores will further alter the refractive index and induce a corresponding spectral shift, enabling direct visual feedback on drug release kinetics.

### Photothermal and drug release performances

**Fig. 3:**
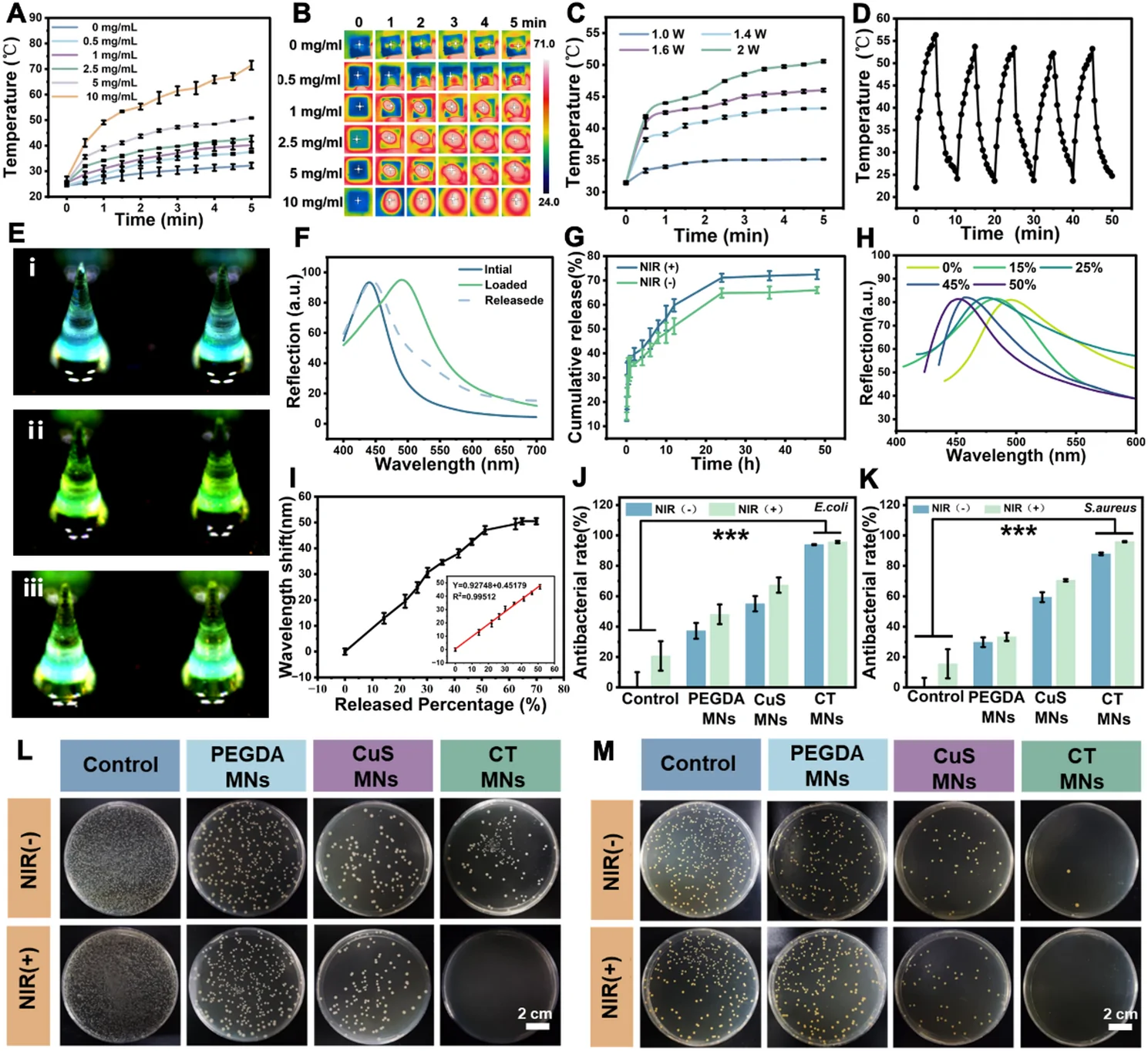
Photothermal, drug release, and antibacterial performances. (**A**) Temperature curves and (**B**) thermal infrared images of CT MNs containing different concentrations of CuS NPs under NIR irradiation (808 nm, 2.0 W/cm^2^); (**C**) Temperature curves of CT MNs upon 808 nm NIR irradiation at varied power densities; (**D**) Temperature profile of CT MNs over five on/off cycles of NIR irradiation (808 nm, 2.0 W/cm^2^); (**E**) Microscopic images and (**F**) Diffraction spectra of CT MNs before drug loading, after drug loading, and after drug release; (**G**) Cumulative release of TB NPs from CT MNs with or without NIR irradiation in PBS (pH 7.4) at 37 ℃; (**H**) Diffraction spectra of CT MNs during the drug release process; (**I**) Calibration curve of diffraction wavelength shift versus cumulative release percentage. Antibacterial activity of CT MNs against (**J**) *E. coli* and (**K**) *S. aureus* assessed by CFU counting; Photographic plates showing bacterial colonies of (**L**) *E. coli* and (M) *S. aureus*. *n* = 3 (biological replicates), data are presented as mean ± SD

To enhance the antibacterial efficacy of the MNs, a synergistic strategy was implemented that combines herbal self-assembly with NIR photothermal activation. The photothermal performance was optimized by irradiating MNs containing varying concentrations of CuS NPs under NIR irradiation (808 nm, 2.0 W/cm^2^). MNs incorporating 5 mg/mL CuS NPs reached a temperature of approximately 50 °C after 5 min of irradiation (Fig. 3A, B), a level sufficient for effective bacterial eradication [24], and were therefore selected for all subsequent studies. Power‑dependent experiments confirmed that irradiation at 2.0 W/cm^2^ for 5 min consistently achieved this target temperature (Fig. 3C), while cyclic on/off irradiation demonstrated excellent photothermal stability over five consecutive cycles (Fig. 3D).

The inverse opal hydrogel scaffold of the IOMNs provides a porous matrix for drug loading. In this study, TB NPs were encapsulated within a 2% agarose solution—a temperature-responsive carrier that gels at room temperature and liquefies above approximately 50 °C—and infused into the nanopores of the IOMNs. This design enables localized drug release primarily at the wound site, accelerated by the temperature increase induced by photothermal effects. The drug release profile of the composite MNs was evaluated in PBS (pH 7.4) at 37 °C. Analysis of the diffraction peaks revealed that after drug loading, the MNs exhibited a red shift, accompanied by a color change from blue to green (Fig. 3E, F). With drug release, a gradual blue shift back toward the original wavelength was observed (Fig. 3F). Besides, in vitro quantification of released TB NPs confirmed that photothermal stimulation accelerated drug release (Fig. 3G).

Beyond controlled release, the colloidal crystal structure of the MNs enables optical monitoring of drug release. Diffraction spectra were recorded at incremental cumulative release levels of 15%, 25%, 45%, and 50%. As shown in Fig. 3H, increasing drug release induced a pronounced blue shift in the diffraction wavelength. A linear calibration curve with a high correlation coefficient (*R*^*2*^ = 0.99512) was established between the wavelength shift and the cumulative release percentage (Fig. 3I), allowing quantitative, non-invasive monitoring of release kinetics. This optical response stems from the decrease in the average refractive index of the hydrogel matrix as TB NPs are released from the pores. The corresponding shift in structural color provides a direct visual indicator of release progress, demonstrating the potential of CT MNs to be developed into an inline, self-reporting drug delivery system for future in vivo applications.

### Antibacterial activity, antioxidant property, and biocompatibility

The antibacterial efficacy of the MN patches was systematically evaluated in vitro against *Escherichia coli* (*E. coli*) *and Staphylococcus aureus* (*S. aureus*). Experiments included four material groups—control (PBS), PEGDA MNs, CuS MNs, and CT MNs—each tested with and without NIR irradiation. The results from both optical density measurement (Fig. S6) and colony-forming unit (CFU) counting (Fig. 3J, K) revealed consistent trends across all experimental groups. Notably, the CuS MNs + NIR group achieved killing rates of 67.35% against *E. coli* and 70.57% against *S. aureus*, while the CT MNs + NIR group showed significantly higher rates of 95.65% and 95.92%, respectively (Fig. 3J, K), confirming the synergistic effect of photothermal therapy and TB NP-mediated antibacterial action. Corresponding colony formation assays (Fig. 3L, M) visually confirmed the pronounced reduction in bacterial viability. These results clearly demonstrate a synergistic mechanism: the photothermal effect of CuS NPs not only directly inactivates bacteria but also potentiates the therapeutic action of the co-assembled TB NPs. Thus, the superior antibacterial activity observed is due to the combined action of the integrated photothermal-chemical strategy and the controlled release of herbal drugs, which work concurrently to enhance microbial clearance.

**Fig. 4:**
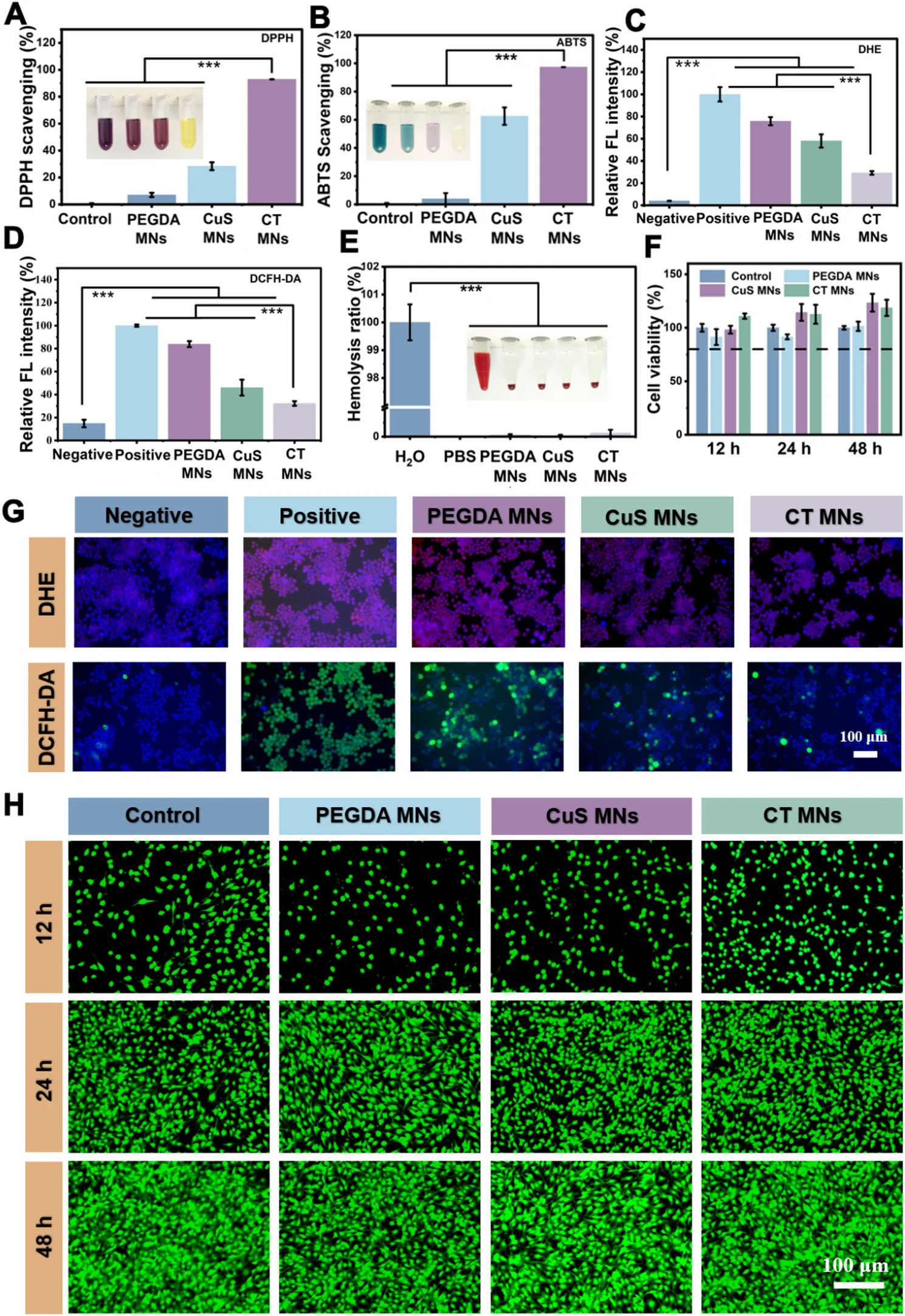
Antioxidant and biocompatibility profiling. (**A**) DPPH scavenging rates and (**B**) ABTS⁺ scavenging rates of different MNs; Relative fluorescence intensity of intracellular (**C**) •O_2_^−^ detected by DHE staining and (**D**) total ROS detected by DCFH-DA staining; (**E**) Hemolysis rates of different MNs; (**F**) Viability of NIH 3T3 cells upon exposure to different MN extracts; (**G**) Fluorescence imaging of intracellular antioxidant stress response (DHE: red = •O_2_^−^; DCFH-DA: green = total ROS); (**H**) Live/dead cell staining of NIH 3T3 cells cultured with different MNs (green: live cells, red: dead cells). *n* = 3 (biological replicates), data are presented as mean ± SD

Excessive production of ROS associated with wound inflammation substantially impairs tissue repair. MNs capable of effectively scavenging surplus ROS and alleviating inflammation held significant potential to accelerate wound healing. The antioxidant potential of the MNs was assessed using DPPH and ABTS⁺ radical scavenging assays. When the MNs reacted with these free radicals, the CT MNs group showed a clear reduction in both solution color (inserts of Fig. 4A, B). Quantitatively, the CT MNs achieved a DPPH scavenging efficiency of 92.93% (Fig. 4A) and an ABTS⁺ scavenging efficiency of 97.36% (Fig. 4B), confirming their potent antioxidant activity and potential to mitigate ROS-mediated inflammation. The antioxidant efficacy was further validated at the cellular level using an oxidative stress model induced in RAW 264.7 cells by 100 µM H_2_O_2_. Intracellular superoxide anion (•O_2_^−^) and total ROS were assessed using DHE and DCFH-DA fluorescent probes, respectively. Fluorescence imaging revealed strong red (DHE) and green (DCFH-DA) signals in the positive group (Fig. 4G), confirming successful ROS induction. Following treatment with CT MNs, the fluorescence intensities decreased markedly to 29.61% (DHE, Fig. 4C) and 32.22% (DCFH-DA, Fig. 4D) relative to the stressed control, demonstrating the ability of CT MNs to restore pathological ROS levels to near-physiological ranges.

Biocompatibility was assessed through hemocompatibility and cytocompatibility studies. Hemolysis assays revealed low hemolytic rates for PEGDA MNs, CuS MNs, and CT MNs, recorded at 0.046%, 0.034%, and 0.130%, respectively (Fig. 4E). All measured values fell well within the safe range, in full compliance with the < 5% hemolysis limit specified in the ISO 10993-4 standard for biomaterials. Cytocompatibility was evaluated using NIH 3T3 fibroblasts cultured with different MN extracts. CCK-8 assays showed a significant proliferative trend in cells treated with CuS MNs and CT MNs over 24 and 48 h (Fig. 4F), attributable to the pro-proliferative effects of the incorporated CuS NPs and TB NPs, which are conducive to cell adhesion, proliferation, and migration. Additionally, live/dead staining confirmed high cell viability throughout the study period, with no observable cell death in MN-treated groups (Fig. 4H). Together, these results confirm the outstanding biocompatibility of the developed CT MNs.

### In vivo wound healing evaluation

**Fig. 5:**
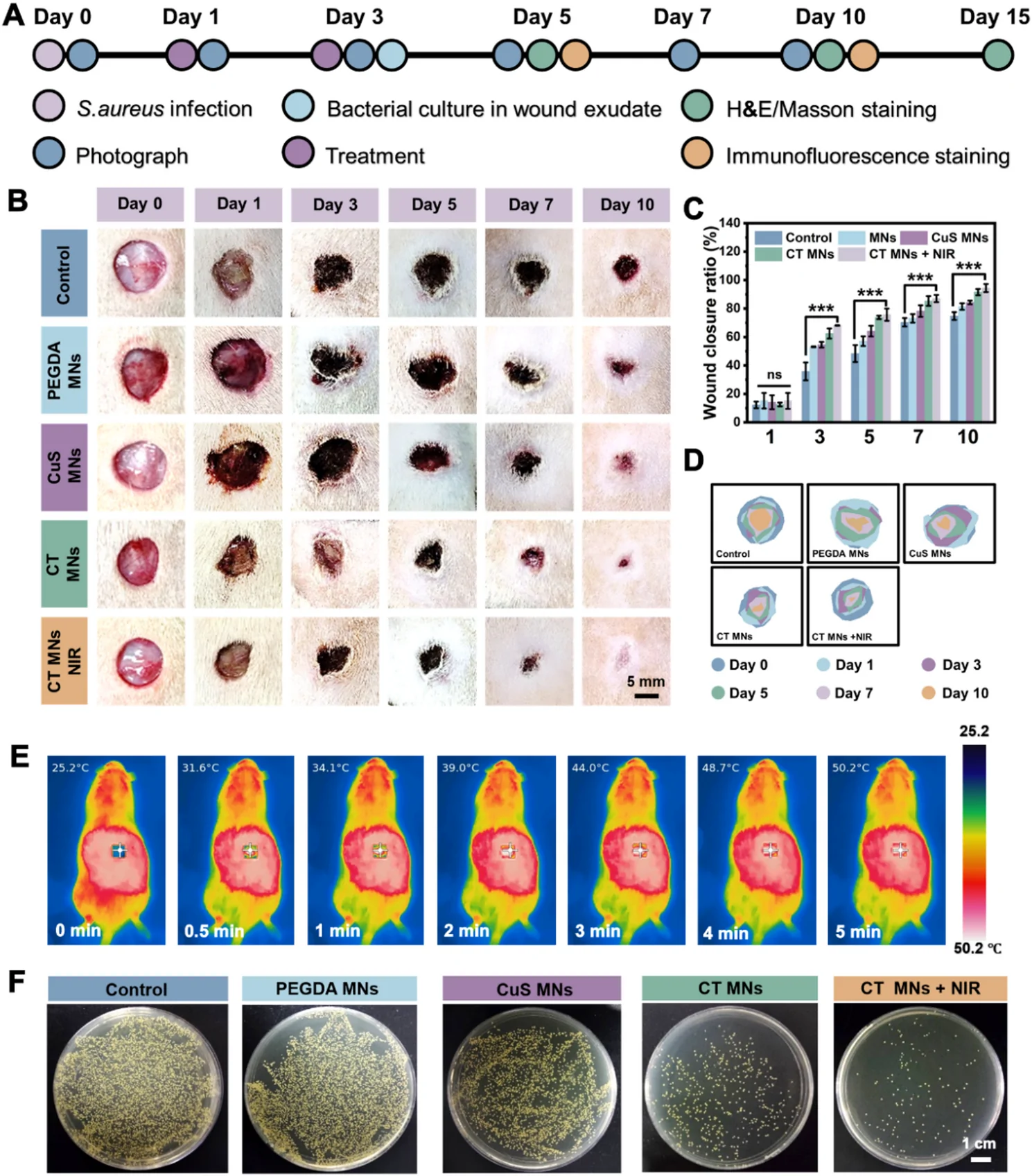
In vivo evaluation of MN‑mediated wound therapy. (**A**) Experimental workflow of the in vivo wound healing study. (**B**) Representative images showing wound area changes on days 0, 1, 3, 5, 7, and 10 post-surgery for different treatment groups. (**C**) Quantitative analysis of wound closure ratios and (**D**) traces of wound closure for different treatment groups post-surgery. (**E**) Thermal images of the wound region for different NIR irradiation times. (**F**) Representative images of bacterial colonies cultured from wound exudate under different treatments. *n* = 4 (animals per group), data are presented as mean ± SD

The therapeutic potential of the MNs was then evaluated in a rat model of *S. aureus*-infected full-thickness skin wounds. Wound closure was monitored from day 0 to day 15 (Fig. 5A). Visual assessment and quantitative analysis using ImageJ software (Fig. 5B-D) demonstrated markedly accelerated healing across all MN-treated conditions relative to the untreated control. As shown in Fig. 5C, the CT MNs + NIR group exhibited the most pronounced healing rate. Within the first five days, this group achieved a wound closure ratio of 75.69%, significantly surpassing the ratios observed in the PEGDA MNs (57.09%), CuS MNs (64.17%), and CT MNs (73.78%) groups, while the control group healed only 48.38%. Notably, even without NIR, CT MNs achieved significantly better wound healing than CuS MNs at day 10 (91.57% vs. 84.31%, *p* < 0.01), directly demonstrating the intrinsic therapeutic activity of TB NPs. Under NIR, this effect was further enhanced by photothermal ablation, leading to 94.41% closure in the CT MNs + NIR group, indicating a strong synergistic effect between TB NP-mediated therapy and photothermal ablation for near-complete wound healing.

The enhanced efficacy of the CT MNs + NIR protocol is attributed to the combined photothermal and antibacterial action. In vivo NIR irradiation induced a rapid temperature increase at the wound site to 50 °C within 5 min (Fig. 5E), enabling immediate photothermal therapy. The antibacterial effect was further quantified by culturing wound exudate collected on day 3. While the control group showed robust bacterial growth, exudate from the CT MNs + NIR group yielded far fewer *S. aureus* colonies (Fig. 5F), confirming potent bacterial clearance. These findings demonstrate that the integration of TB NPs with NIR-mediated photothermal therapy synergistically controls infection, shortens the healing period, and minimizes scar formation, underscoring its significant therapeutic potential for infected wound management.

### In vivo wound histological evaluation

**Fig. 6:**
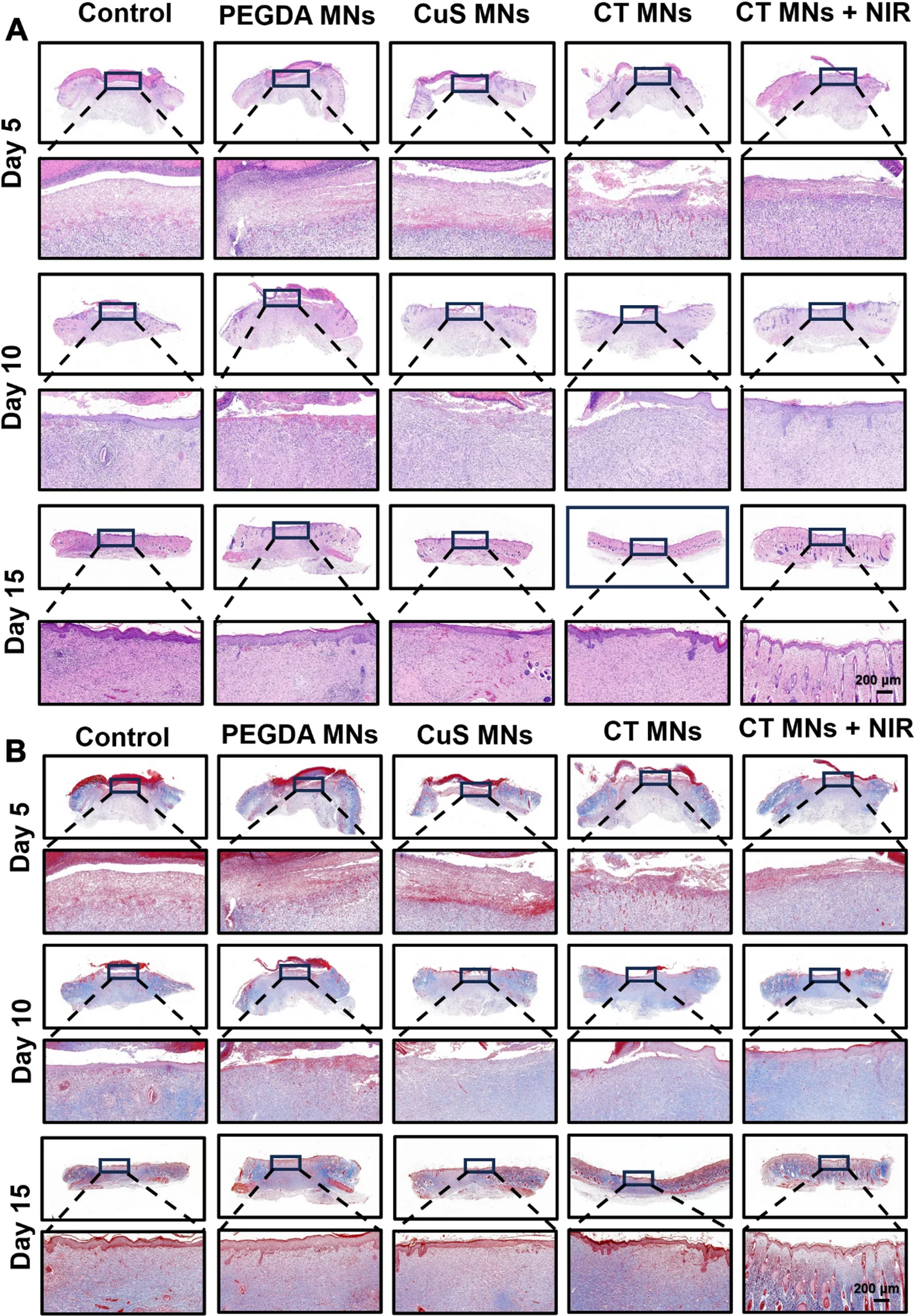
Histological evaluation of regenerated skin tissue at postoperative days 5, 10, and 15. (**A**) H&E staining. (**B**) Masson’s trichrome staining. *n* = 3 (biological replicates)

A comprehensive histopathological evaluation using hematoxylin and eosin (H&E) and Masson’s trichrome staining was conducted to assess tissue regeneration within the infected wound microenvironment. On postoperative day 5, the control group exhibited significant neutrophil infiltration and extensive scab formation (Fig. 6A), reflecting persistent inflammatory damage, whereas the CT MNs + NIR group showed markedly reduced inflammation with minimal scabbing. Importantly, no signs of thermal necrosis or severe inflammation were observed in the CT MNs + NIR group, confirming that the NIR irradiation parameters (808 nm, 2.0 W/cm^2^, 5 min, ~ 50 °C) are safe for in vivo application. By day 10, the CT MNs + NIR treatment further promoted tissue regeneration, characterized by a pronounced increase in nascent capillary density and the formation of a continuous basement membrane. Masson’s trichrome staining (Fig. 6B) confirmed enhanced collagen deposition across all treatment groups, with the CT MNs + NIR group displaying a well-organized, mesh-like collagen architecture, and a fiber diameter distribution shifting toward physiological norms. Collectively, these results demonstrate that the CT MNs + NIR system effectively suppresses early inflammation and orchestrates structured collagen remodeling, thereby chronologically facilitating integrated wound repair in an infected setting.

**Fig. 7:**
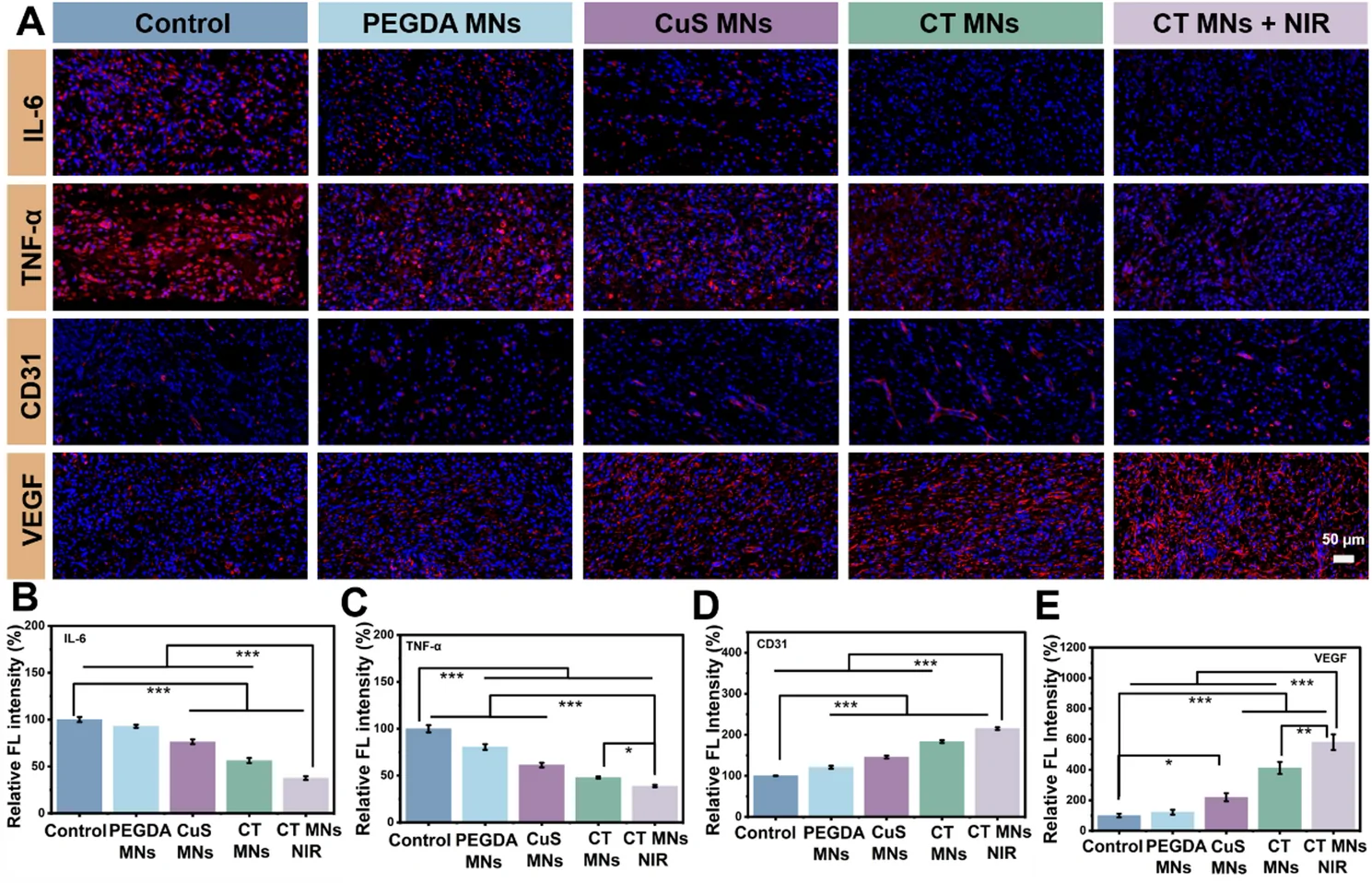
Immunofluorescence analysis of wound healing markers. (**A**) Immunofluorescence staining of wound tissues for inflammatory cytokines (day 5: IL-6, TNF-α) and angiogenesis markers (day 10: CD31, VEGF); All signals shown in red, and uuclei were counterstained with DAPI (blue); Quantification of the expression levels of (**B**) IL-6, (**C**) TNF-α, (**D**) CD31, and (**E**) VEGF in healing skin. *n* = 3 (image fields), data are presented as mean ± SD

The precise regulation of inflammation and angiogenesis is critical for effective wound healing. To investigate the immunomodulatory effect of the MN system, the expression of key pro‑inflammatory cytokines—interleukin‑6 (IL‑6) and tumor necrosis factor‑alpha (TNF‑α)—was analyzed via immunofluorescence imaging. On postoperative day 5, wounds treated with CT MNs + NIR exhibited markedly weaker red fluorescence signals for both IL‑6 and TNF‑α in comparison to the control group (Fig. 7A). Semi‑quantitative analysis further confirmed a significant reduction in the expression levels of IL‑6 (Fig. 7B) and TNF‑α (Fig. 7C), indicating a pronounced ability of the CT MNs + NIR treatment to suppress excessive inflammatory responses. In parallel, the angiogenic capacity was assessed by monitoring the expression of platelet endothelial cell adhesion molecule-1 (CD31) and vascular endothelial growth factor (VEGF). The CuS MNs group (without NIR irradiation) exhibited higher CD31 and VEGF expression compared to the PEGDA MNs group (Fig. 7A, D and E), suggesting a possible pro-angiogenic effect associated with CuS NPs and, by extension, Cu^2+^. Furthermore, the CT MNs + NIR group showed upregulated expression of both markers as early as day 10, and maintained significantly higher levels of these neovascularization indicators relative to the control, which can be attributed to the comprehensive effects of antibacterial, anti-inflammatory, and tissue-repairing functions of the system. Together, these data illustrate that the CT MNs + NIR system coordinately downregulates early inflammation and enhances subsequent vascularization, thereby creating a regenerative microenvironment conducive to accelerated wound repair.

## Conclusion

In this study, we developed and validated a CT MNs patch for infected wound management that uniquely integrates NIR-triggered photothermal therapy, antioxidant/antibacterial activity, and real-time drug release monitoring. The system achieves potent antibacterial effects, ROS elimination, and pro-angiogenic activity, collectively promoting wound regeneration. A key innovation is the incorporation of an inverse opal structure, which enables visual feedback through quantifiable blue shifts in structural color corresponding to drug release. In vivo, the CT MNs + NIR group achieved 94.41% wound closure by day 10, outperforming all controls. As a proof-of-concept study, this work has some limitations that should be acknowledged, particularly regarding the detailed mechanistic understanding of angiogenesis and the comprehensive in vivo assessment of synergistic effects. These aspects are critical and will be systematically addressed in our subsequent research. Despite these limitations, this work establishes a multifunctional platform that merges targeted therapy with autonomous visual monitoring, offering a promising strategy for intelligent wound care. Future studies will focus on quantitative mechanistic validation and clinical translation.

## Supplementary Information

Supplementary Material 1.

## Data Availability

Data will be made available on request.
