# Supplementary material for: Structural color-based microneedle patch with NIR-triggered on-demand drug release for intelligent treatment of infected wounds
Source: J Nanobiotechnology. 2026 Jun 27;24:864. doi: 10.1186/s12951-026-04730-6 (PMC13573378; doi:10.1186/s12951-026-04730-6)
Supplement: Supplementary file 1 [file 12951_2026_4730_MOESM1_ESM.docx]

**Supplementary Information**

**Structural color-based microneedle patch with NIR-triggered on-demand drug release for intelligent treatment of infected wounds**

Sijia Huang ^1^, Menglan Yang ^1^, Chen Ding ^1^, Wenfang Du ^2, *^, Shengyuan Yang ^1, *^, Qiang Xi ^1, 3 *^, Fubing Xiao ^1, *^

^1^Hunan Prevention and Treatment Institute for Occupational Diseases, Affiliated Prevention and Treatment Institute for Occupational Diseases of University of South China, School of Public Health, Hengyang Medical School, University of South China, Changsha 410007, China.

^2^School of Chemical and Environmental Engineering, Hunan Institute of Technology, Hengyang 421002, China.

^3^Laboratory for the Development and Application of New Technologies in the Diagnosis and Treatment of Chemical Poisoning, Hunan Provincial Clinical Medical Research Center for Occupational Diseases, Changsha 410007, China.

* Corresponding author.

*E-mail address: duwenfang20@163.com* (Wenfang Du)

*yangshyhy@126.com* (Shengyuan Yang)

*xiqiang@hnu.edu.cn* (Qiang Xi)

*xiaofubing2008@163.com* (Fubing Xiao)

**Contents**

**Additional experimental section**

**S-3.** Synthesis of CuS NPs.

**S-3.** Synthesis of TB NPs.

**S-3.** Synthesis of SiO₂ nanoparticles.

**S-4.** Preparation of colloidal crystal MN templates.

**S-4.** DPPH radical scavenging assay.

**S-4.** ABTS^+^ radical scavenging assay.

**S-5.** Assessment of intracellular oxidative stress scavenging capacity.

**S-7.** Hemolysis test.

**S-7.** Experimental animal feeding.

**S-7.** *In vivo* wound histological evaluation.

**S-8.** Measurements.

**S-8.** Statistical analysis.

**Figures**

**S-10.** Fig. S1. (A) Standard calibration curve for TB NPs quantification; (B) UV absorption spectra of different samples.

**S-11.** Fig. S2. Characterization of nanoparticles.

**S-12.** Fig. S3. SEM characterization of MNs.

**S-13.** Fig. S4. SEM imaging and elemental mapping of MNs.

**S-14.** Fig. S5. Characterization of MNs mechanical and penetration properties.

**S-15.** Fig. S6. Antibacterial activity of MNs against A) *E. coli* and B) *S. aureus* assessed by optical density (OD_600_) measurement.

**Additional experimental section**

**Synthesis of CuS NPs:** 5 mL CuCl_2_·2H_2_O solution (20 mM) was mixed with 100 μL polyvinylpyrrolidone (PVP, 60 mg/mL) in 20 mL deionized water, followed by magnetic stirring at room temperature for 30 min. Then, 50 μL Na_2_S solution (2 M) was added dropwise, followed by stirring for 5 min. Following the reaction at 90 °C for 15 min in an oil bath and subsequent cooling to room temperature, the mixture was centrifuged at 10,000 rpm for 10 min. The resulting product was washed three times with deionized water, and vacuum-dried for 12 h to obtain CuS NPs.

**Synthesis of TB NPs:** Berberine (BBR) was precisely weighed and dissolved in anhydrous ethanol to prepare a 1 mg/mL stock solution. Tannic acid (TA) was dissolved in deionized water to prepare a 4.5 mg/mL solution, followed by ultrasonication for dispersion. The BBR ethanol solution was added dropwise to the TA aqueous solution using a micro-syringe. The mixture was then magnetically stirred at room temperature for 2 h to facilitate molecular self-assembly. The reaction solution was dialyzed using a membrane (molecular weight cutoff: 3.5 kDa) to remove free molecules. The resulting product was subsequently lyophilized to obtain TB NPs.

**Synthesis of SiO_2_ nanoparticles:** A mixture of 130 mL ethanol, 36 mL deionized water, 14 mL ammonia, and a specific volume of the 18 nm SiO_2_ seed solution was added to a 250 mL conical flask. The mixture was stirred at 50 °C in a water bath for 5 min using a magnetic stirrer. Subsequently, 10 mL tetraethyl orthosilicate (TEOS) was added, and the reaction continued under the same conditions (50 °C) for 3 h. The solution was centrifuged at 7,000 rpm, washed three times, and dried to obtain SiO_2_ nanoparticles. The final particle size was controlled by adjusting the seed solution volume.

**Preparation of colloidal crystal MN templates****:** Colloidal crystal MN templates were fabricated using a sedimentation method. A 5% (w/v) SiO_2_ ethanol solution was ultrasonicated for 5 min to ensure uniform dispersion and then injected into a hydrophilized polydimethylsiloxane (PDMS) microneedle mold. Vacuum (-0.1 MPa) was applied to remove bubbles, and the mold was left undisturbed for 12 h to allow natural sedimentation and drying, yielding an ordered SiO_2_ colloidal crystal MN template.

**DPPH radical scavenging assay:** The experimental groups included: PEGDA MNs, CuS MNs, and CT MNs. The PBS-treated group served as the control. A DPPH (2,2-diphenyl-1-picrylhydrazyl) solution was prepared by dissolving DPPH in anhydrous ethanol and diluted to an optical density at 517 nm (OD_517_) of 0.8-1.0. Subsequently, 5 mL of this DPPH solution was incubated with the MNs for 45 min at 37 °C, protected from light. The absorbance at 517 nm was then measured. The DPPH radical scavenging activity was calculated using the following formula: DPPH scavenging rate (%) = (*A_0_* − *A_1_*) / *A_0_* × 100% where *A_0_* and *A_1_* represent the absorbance of the control and test groups, respectively (*n* = 3, biological replicates).

**ABTS^+^ radical scavenging assay:** The ABTS⁺ (2,2'-azino-bis(3-ethylbenzothiazoline-6-sulfonic acid) radical cation was generated by mixing 7 mM ABTS⁺ stock solution with 2.45 mM potassium persulfate solution in equal volumes. The mixture was allowed to react in the dark for 12 h. The resulting ABTS⁺ working solution was diluted to an appropriate absorbance. Then, 5 mL of the ABTS⁺ solution was incubated with the MNs for 30 min at 37 °C, protected from light. The absorbance at 734 nm was measured. The ABTS⁺ radical scavenging activity was calculated as: ABTS⁺ scavenging rate (%) = (*A_0_* − *A_1_*) / *A_0_* × 100%, where *A_0_* and *A_1_* represent the absorbance of the control (ABTS⁺ solution incubated with PBS) and test groups, respectively (*n* = 3, biological replicates).

**Assessment of intracellular oxidative stress scavenging capacity:** This study quantified intracellular ROS levels using fluorescent probes: 2',7'-dichlorofluorescin diacetate (DCFH-DA) for general ROS and dihydroethidium (DHE) for specific detection of superoxide anion (•O_2_^-^). The detailed experimental procedure is as follows: RAW 264.7 macrophages were seeded in 12-well plates at a density of 5 × 10^4^ cells per well. The cells were pre-cultured in a constant temperature incubator at 37 °C with 5% CO_2_ for 12 h until reaching a semi-adherent state. To simulate an oxidative stress environment, the pre-cultured cells were stimulated with a 100 μM of hydrogen peroxide (H_2_O_2_) solution for a duration of 6 h, thereby establishing the oxidative stress model.

This study systematically evaluated the antioxidative stress performance of MNs by designing different experimental groups to comprehensively investigate their regulatory effects on oxidative stress. The groups were specifically divided into: Negative control group (untreated cells); Positive control group (100 μM H_2_O_2_ induced treatment group, to assess if oxidative stress was successfully established); PEGDA MNs group (100 μM H_2_O_2_ induced group treated with PEGDA MNs extract solution); CuS MNs group (100 μM H_2_O_2_ induced group treated with CuS MNs extract solution); CT MNs group (100 μM H_2_O_2_ induced group treated with CT MNs extract solution).

These five groups were labeled as Negative, Positive, PEGDA MNs, CuS MNs, and CT MNs, respectively. The corresponding extract solutions for each experimental group were added to the aforementioned 12-well plates and co-cultured with the cells for 12 h. After gently washing three times with PBS (pH 7.4) to remove the stimulus, 5 μM working solutions of the fluorescent probes were added separately. Incubation was performed at 37 °C in the dark for 30 min to complete probe labeling. Subsequently, PBS was used again for three thorough washes to remove unbound probes. Following this, 5 μM of DAPI staining solution was added for nuclear localization staining in the dark for 10 min.

Fluorescence imaging was performed using an inverted fluorescence microscope (Nikon DS-Ri2, Japan) to capture images of each channel: DHE: excitation/emission wavelength = 518/605 nm (red fluorescence); DCFH-DA: excitation/emission wavelength = 504/529 nm (green fluorescence); DAPI: excitation/emission wavelength = 358/461 nm (blue fluorescence); Finally, semi-quantitative analysis of fluorescence intensity was performed using ImageJ software. To ensure the reliability of the experimental results and statistical significance, at least three independent fields of view were captured per experimental group for statistical analysis.

**Hemolysis test**: The study evaluated the blood compatibility of CT MNs through an *in* *vitro* hemolysis assay. The experiment included three MNs sample groups (PEGDA MNs, CuS MNs, CT MNs), with PBS solution as the negative control and deionized water as the positive control. The specific operational procedure is as follows: anticoagulated whole blood was subjected to gradient centrifugation (1,500 rpm, 10 min) to separate red blood cells (RBCs). The isolated RBCs were diluted 5-fold with physiological saline to prepare an RBC suspension. Subsequently, 800 μL of the suspension was co-incubated with each MNs sample (37 °C, 1 h). After centrifugation of the reaction system (1500 r/min, 10 min), the supernatant was collected, and its absorbance was measured at 545 nm. The hemolysis ratio was calculated according to the following formula: Hemolysis ratio (%) = (*A_s_* −*A_nc_*) / (*A_pc_* − *A_nc_*) × 100%, where *A_s_*, *A_pc_*, and *A_nc_* represent the absorbance values of the sample, positive control, and negative control, respectively.

**Experimental animal feeding:** Adult male Sprague Dawley (SD) rats (210 - 250 g) were housed in the Department of Laboratory Animal Science of the University of South China. The photoperiod was maintained at 12 h of light and 12 h of dark, the room temperature was 18 - 22°C, and the humidity was controlled between 50 - 60%. The rats had free access to drinking water and food. All experimental animal procedures were approved by the Laboratory Animal Ethics Committee of University of South China.

***In vivo* wound histological evaluation:** Collected specimens were fixed in 4% neutral buffered formalin for 24 h, then processed according to standard protocols for hematoxylin and eosin (H&E) and Masson's trichrome staining; whole-slide images were acquired using a digital pathology slide scanning system and subjected to histological analysis; Immunofluorescence techniques were employed to stain inflammatory factors (TNF-α; IL-6) and angiogenesis markers (CD31; VEGF) within the wound tissue, and the fluorescence signal intensity was semi-quantitatively analyzed using ImageJ software after statistical processing.

**Measurements:** The morphology was analyzed using a scanning electron microscope (SEM, TESCAN MIRA LMS, Czech Republic) and a transmission electron microscope (TEM, Carl Zeiss, Germany). The particle size and zeta potential of nanoparticles were determined using a Zeta potential and particle size analyzer (Zetasizer Pro, Malvern, UK). The UV absorption peak of the nanoparticles was characterized using a UV-Vis spectrophotometer (UV-2550, Shimadzu, Japan). The structure of TB NPs was characterized by ^1^H NMR spectroscopy (AVANCE NEO 500 MHz, Bruker, Germany). Crystallinity was examined via X-ray diffraction (XRD, Rigaku Smartlab SE, Japan). Mechanical properties were measured with an electronic universal testing machine (CMT6103, China Meters Industrial System).

**Statistical analysis:** Data analysis was performed using GraphPad Prism 8.0 (GraphPad Software, San Diego, CA), and results were expressed in the form of mean ± standard deviation (SD); *In vitro* experiments were performed with three biological replicates (*n* = 3); for *in vivo* wound healing study, 4 SD rats were used in each group (*n* = 4). One-way analysis of variance (one-way ANOVA) followed by Tukey's test was employed; significance was defined as: **P* < 0.05; ***P* < 0.01; ****P* < 0.001; NS indicates no significant difference (*P* ≥ 0.05).

**Fig. S1** (**A**) Standard calibration curve for TB NPs quantification; (**B**) UV absorption spectra of different samples.

**Fig. S2** Characterization of nanoparticles. Dynamic light scattering (DLS) analysis of (**A**) CuS NPs and (**B**) TB NPs; (**C**) ^1^H NMR spectra of TA, BBR, and TB NPs.

**Fig. S3** SEM characterization of SiO_2_ colloidal crystal template, colloidal crystal MNs, and IOMNs (inverse opal microneedles). (**A**) SEM image of the SiO_2_ colloidal crystal template; (**B**) SEM image of the colloidal crystal MNs; (**C**) Cross-sectional SEM image of the IOMNs; (**D**) SEM image of the MN tip array; (**E**) SEM image of a single colloidal crystal MN tip; (**F**) Magnified SEM view of a single colloidal crystal MN tip; (**G**) SEM image of a single IOMN tip; (**H**) Magnified SEM view of a single IOMN tip.

**Fig. S4** SEM images, elemental mapping, and EDS spectra of colloidal crystal MNs and IOMNs. SEM images and corresponding elemental mapping of (**A**) colloidal crystal MNs and (**B**) IOMNs; EDS spectra of (**C**) colloidal crystal MNs and (**D**) IOMNs.

**Fig. S5** Mechanical properties and skin penetration performance of MNs. (**A**) Force-distance curves; (**B**) Macroscopic image of MNs; (**C**) Penetration effect of MNs in rat skin; (**D**) Histological cross-section of rat skin after MNs penetration; (**E**) Magnified histological image of the penetration site.

**Fig. S6** Antibacterial activity of MNs against (**A**) *E. coli* and (**B**) *S. aureus* assessed by optical density (OD_600_) measurement.
